# Plasma Concentrations of Vinculin versus Talin-1 in Coronary Artery Disease

**DOI:** 10.3390/medsci10030046

**Published:** 2022-08-26

**Authors:** Masayuki Aoyama, Yoshimi Kishimoto, Emi Saita, Reiko Ohmori, Kojiro Tanimoto, Masato Nakamura, Kazuo Kondo, Yukihiko Momiyama

**Affiliations:** 1 Department of Cardiology, NHO Tokyo Medical Center, Tokyo 152-8902, Japan; 2 Toho University Graduate School of Medicine, Tokyo 153-8515, Japan; 3 Department of Food Science and Human Nutrition, Faculty of Agriculture, Setsunan University, Osaka 573-0101, Japan; 4 Faculty of Human Life and Environmental Sciences, Ochanomizu University, Tokyo 112-8610, Japan; 5 Faculty of Regional Design, Utsunomiya University, Tochigi 321-8505, Japan

**Keywords:** coronary artery disease, talin-1, vinculin

## Abstract

Vinculin and talin-1, which are cytoskeletal proteins affecting focal adhesions, were reported to be down-expressed in atherosclerotic lesions. Recently, we reported high concentrations of plasma talin-1 in patients with coronary artery disease (CAD). However, blood vinculin concentrations in CAD patients have not been clarified. Plasma vinculin concentrations as well as talin-1 were studied in 327 patients in whom coronary angiography was performed. CAD was proven in 177 patients (1-vessel, *n* = 79; 2-vessel, *n* = 57; 3-vessel disease, *n* = 41). However, vinculin concentrations were not markedly different between the CAD(-) and CAD groups (median 122.5 vs. 119.6 pg/mL, *p* = 0.325) or among patients with CAD(-), 1-, 2-, and 3-vessel diseases (122.5, 112.8, 107.9, and 137.2 pg/mL, *p* = 0.202). In contrast, talin-1 concentrations were higher in CAD than the CAD(-) group (0.29 vs. 0.23 ng/mL, *p* = 0.006) and increased stepwise in the number of stenotic vessels: 0.23 in CAD(-), 0.28 in 1-vessel, 0.29 in 2-vessel, and 0.33 ng/mL in 3-vessel disease (*p* = 0.043). No correlation was observed between vinculin and talin-1 concentrations. In multivariate analysis, vinculin concentrations were not a factor for CAD. In conclusion, plasma vinculin concentrations in patients with CAD were not high and were not associated with the presence or severity of CAD.

## 1. Introduction

Focal adhesion, which is the key attachment of cells and extra-cellular matrix (ECM), plays a crucial role in retaining intercellular communication as well as cell integrity. Integrins are transmembrane glycoprotein receptors that are important for retaining focal adhesions and the adhesive interactions between cells and ECM [1,2]. Talin-1 is an actin-binding protein, which can bind integrins to actin cytoskeleton [1,2,3]. Talin-1 is known as a potent integrin activator affecting cell adhesions and apoptosis [1,4]. Moreover, talin-1 accelerates cell proliferation with cell cycle progression by activating integrin adhesions and focal adhesion proteins [5].

**Figure 1:**
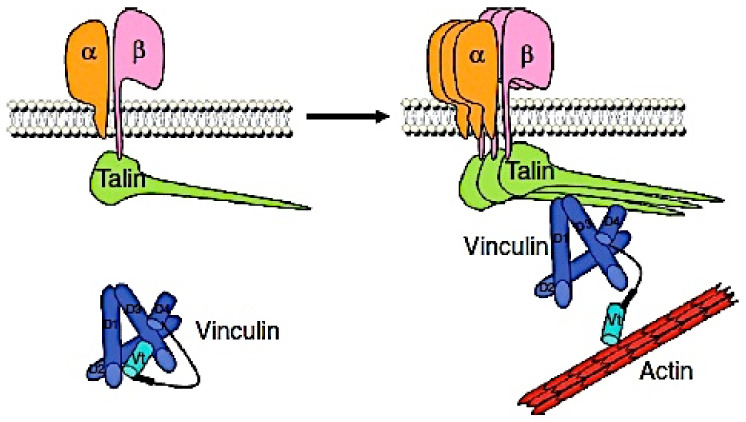
The vinculin and talin-1 interaction regulate integrin clustering at focal adhesions (This figure was adapted from Peng X et al. [11]).

Vinculin is a 117-kDa actin-binding cytoskeletal protein, consisting of the vinculin head, linker, and tail, that connects the cell–ECM and cell–cell adhesions to actin cytoskeleton as a multi-protein linker [6,7,8]. Vinculin is located and enriched at focal adhesions, and, in its absence, the cell–ECM and cell–cell adhesions and their strength are much impaired, indicating a major role of vinculin in human physiology [9,10]. Vinculin also regulates integrin-mediated cell adhesion, migration, and apoptosis, and it can decrease cell motility through its effect on cell adhesion [6,9,11]. Vinculin binds to talin-1 at 22 vinculin-binding sites [12], and the interaction of vinculin with talin-1 is recognized to be important to regulate focal adhesions [6,13]. Vinculin binding to talin-1 results in talin-1 recruitment to the plasma membrane [14]. Moreover, the recruitment of vinculin to talin-1 stabilizes focal adhesions and promotes integrin clustering, and vinculin regulates integrin activation through talin-1 [10,11] (Figure 1). Besides modulating talin-1 activity, vinculin stabilizes integrins by the linkage to actin cytoskeleton [10].

The changes in the cytoskeleton, such as disorganization and modifications in cell–ECM and cell–cell adhesions, are associated with atherosclerosis progression [15,16]. Recently, both vinculin and talin-1 were shown to be down-expressed in atherosclerotic lesions [17]. However, we have recently reported high concentrations of plasma talin-1 in patients with coronary artery disease (CAD) [18]. Because the interaction of vinculin with talin-1 is recognized to be important for regulating focal adhesion, and because vinculin regulates integrin activation through talin-1 [6,10,13], we hypothesized that blood vinculin concentrations would be a better biomarker for CAD than talin-1 concentrations. However, blood vinculin concentrations in CAD patients and any association between blood vinculin and talin-1 concentrations have not been elucidated. Therefore, we extend our previous report [18] by measuring plasma vinculin concentrations and by comparing them with talin-1 concentrations in 327 patients in whom coronary angiography was performed.

## 2. Materials and Methods

### 2.1. Study Population

In our previous study [18], plasma talin-1 concentrations were measured in 349 consecutive patients who underwent elective coronary angiography for suspected CAD at NHO Tokyo Medical Center from June 2009 to September 2016. The institutional review board approved the study (R08-050/R15-056). After taking written informed consent, overnight-fasting blood samples were obtained on the day of the angiography. Patients with acute coronary syndrome (ACS), heart failure, severe valvular heart disease, or any history of coronary artery bypass surgery (CABG) or intervention were excluded. Because patients with cancer had high concentrations of plasma talin-1 [19], we excluded such patients. Moreover, among the 349 study patients, 22 patients were excluded, because their blood samples were insufficient. As a result, plasma vinculin concentrations were measured in the remaining 327 patients. Hypertension was defined as blood pressure (BP) ≥ 140/90 mmHg or taking antihypertensive drugs; in total, 193 (59%) were on medication. Hypercholesterolemia was defined as LDL-cholesterol concentration > 140 mg/dl or taking statin; in total, 121 (37%) were on statin. We defined diabetes mellitus (DM) as fasting glucose concentration ≥ 126 mg/dl or on anti-diabetic medication or insulin, and DM was present in 82 (25%). Because the pack-year but not the period of cessation were reported to have an important correlation with CAD severity [20], we defined smoking as ≥10 pack-years, as used in our and other previous studies [21,22], and 116 patients (35%) were found to be smokers.

### 2.2. Measurements of Vinculin, Talin-1, and C-Reactive Protein (CRP) Concentrations

Blood samples taken with EDTA tubes were centrifuged at 2000× *g* at 4 °C for 15 min. Plasma samples were frozen and stored until use at −80 °C. For the measurement of plasma vinculin concentrations, the frozen plasma was thawed, and then an enzyme-linked immunosorbent assay (ELISA) (Human Vinculin ELISA Kit, CUSABIO, Houston, TX, USA) was used. Its detection range was 28.0 to 1800 pg/mL. The intra- and inter-assay coefficients of variation (CV) were <8% and <10%, respectively. As we previously reported [18], we measured talin-1 concentrations by ELISA (Human TLN ELISA Kit, Elabscience, Houston, TX, USA). Its detection range was 0.10 to 10 ng/mL. The intra- and inter-assay CV were <10% and <10%, respectively. High-sensitivity CRP concentrations were assessed using nephelometry (Dade-Behring BNII instrument).

### 2.3. Angiographic Analysis

We performed coronary angiography (Philips Electronics Japan), and angiographic data were evaluated blindly by a single cardiologist. CAD was defined as ≥1 coronary artery with more than 50% stenosis. As in our previous reports [18,23], the severity of CAD was assessed as the numbers of vessels and segments with >50% stenosis. The coronary artery was classified into 29 segments by CASS classification.

### 2.4. Statistical Analysis

Statistical analysis was performed with IBM software (SPSS version 25, IBM, Tokyo, Japan), and *p*-value < 0.05 was considered statistically significant. Continuous parameters were expressed as the mean ± standard deviation. Categorical parameters were expressed as the number (%). Because vinculin, talin-1, and CRP concentrations were highly skewed and nonparametric, these were expressed as the median and interquartile range. For differences between two groups, an unpaired *t*-test and a Mann–Whitney U test were utilized for parametric and nonparametric parameters. For differences among ≥3 groups, ANOVA with the Scheffe’s test and Kruskal–Wallis test was utilized for parametric and nonparametric parameters, respectively. For categorical parameters, the χ^2^ test was utilized. Correlations between vinculin concentrations and talin-1 or CRP concentrations or CAD severity were also analyzed using the Spearman’s rank correlation test. Regarding the cutoff point of talin-1 for CAD, the ROC curve analysis showed it to be 0.28 ng/mL.

## 3. Results

**Figure 2:**
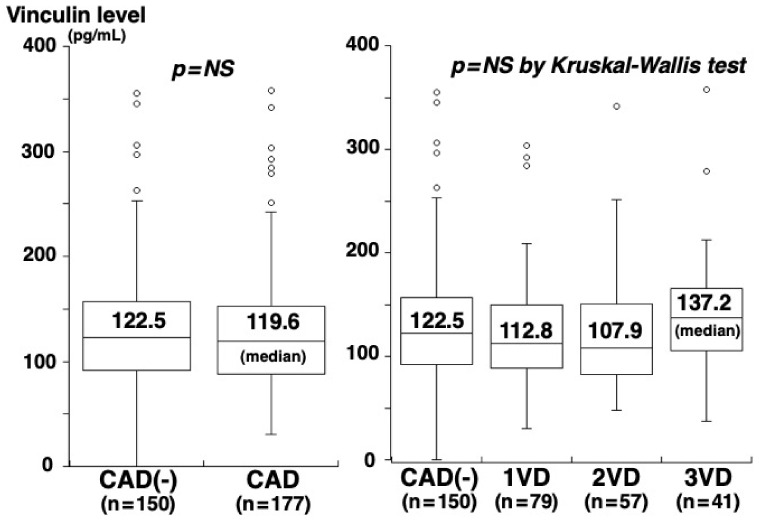
Plasma vinculin concentrations and CAD or the number of vessels with >50% stenosis. Vinculin concentrations were not different between the CAD(-) and CAD groups (*p* = 0.325). Moreover, there was no significant difference in vinculin concentrations among the four groups of CAD(-), 1VD, 2VD, or 3VD (*p* = 0.202). The central line shows the median, and the box shows the 25th to 75th percentiles.

**Figure 3:**
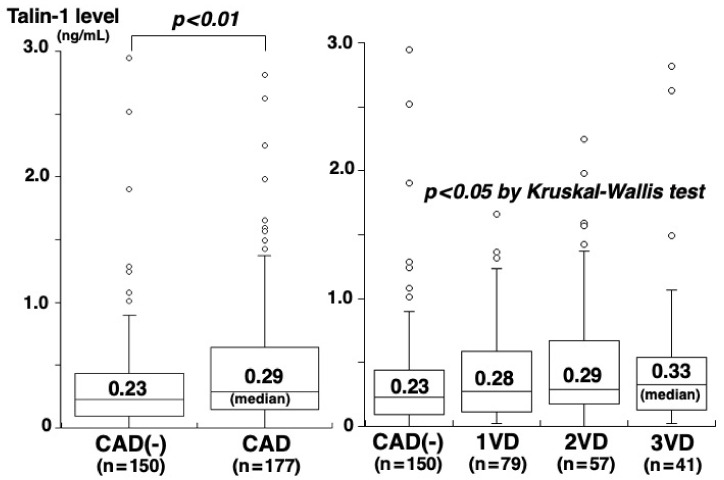
Plasma talin-1 concentrations and CAD or the number of vessels with >50% stenosis. CAD, especially the 3VD group, had higher talin-l concentrations than the CAD(-) group (*p* < 0.01).

Of the 327 study patients, CAD was angiographically proven in 177 (54%) (1-vessel [1VD], *n* = 79; 2-vessel [2VD], *n* = 57; 3-vessel disease [3VD], *n* = 41). The clinical characteristics of patients with and without CAD are shown in Table 1. Patients with CAD had higher CRP concentrations than those without CAD (0.58 vs. 0.43 mg/L, *p* = 0.001) (Table 1). However, no significant difference was found in vinculin concentrations between patients with and without CAD (119.6 vs. 122.5 pg/mL, *p* = 0.325) or among the four groups of CAD(-), 1VD, 2VD, and 3VD (122.5, 112.8, 107.9, and 137.2 pg/mL, *p* = 0.202) (Figure 2). Vinculin concentrations did not correlate with the number of segments with >50% stenosis (rs = 0.03, *p* = 0.635). In contrast, plasma talin-1 concentrations in patients with CAD were higher than those without CAD (0.29 vs. 0.23 ng/mL, *p* = 0.006) and increased on the number of vessels with >50% stenosis: 0.23 in CAD(-), 0.28 in 1VD, 0.29 in 2VD, and 0.33 ng/mL in 3VD (*p* = 0.043) (Figure 3). Talin-1 concentrations correlated with the number of segments with >50% stenosis (rs = 0.13, *p* = 0.018).

**Figure 4:**
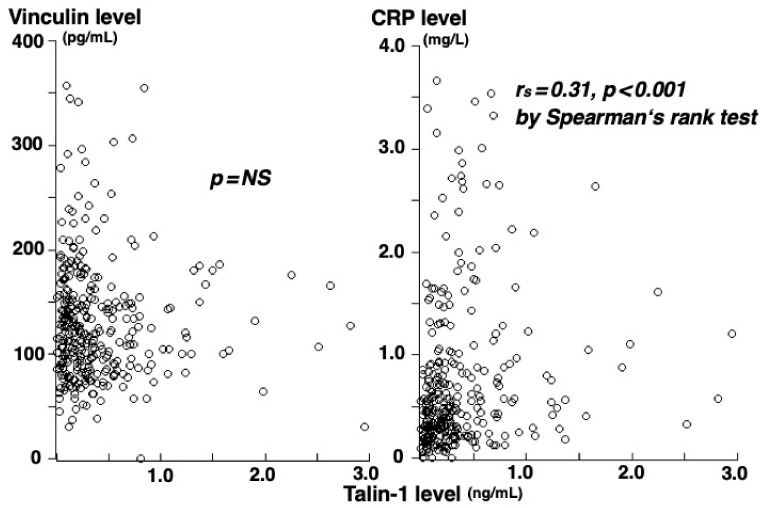
Correlations of plasma talin-1 concentrations with vinculin or CRP concentrations. No correlation was found between vinculin and talin-1 concentrations (rs = −0.06, *p* = 0.308) (**left**). However, talin-1 concentrations correlated with CRP concentrations (rs = 0.31, *p* < 0.001) (**right**).

There was no significant correlation of vinculin concentrations with talin-1 or CRP concentrations, but talin-1 correlated with CRP concentrations (rs = 0.31, *p* < 0.001) (Figure 4). To elucidate the independent association of CAD with vinculin or talin-1 concentrations, variables (atherosclerotic risk factors, CRP, and vinculin and talin-1 concentrations) were included in the multivariate analysis. Talin-1 concentrations were an independent factor for CAD, but vinculin concentrations were not associated. The odds ratio for the presence of CAD was 1.88 (95% confidence interval: 1.15–4.08) for high talin-1 concentration (>0.28 ng/mL) (*p* = 0.012).

## 4. Discussion

The results of our analyses show that vinculin concentrations were not significantly different between patients with and without CAD and did not correlate with CAD severity. No correlation was found between vinculin concentrations and talin-1 or CRP concentrations. However, talin-1 concentrations in patients with CAD were higher than those without CAD and correlated with CAD severity and CRP concentrations.

The cytoskeleton disorganization and modification in the cell–ECM and cell–cell adhesions are recognized as alterations associated with atherosclerosis [15,16]. Both vinculin and talin-1 were shown to be down-expressed in human atherosclerotic lesions, thereby leading to the loosing of cell–ECM interactions [17]. In a rabbit model, vinculin was also shown to be down-expressed in atherosclerotic lesions [15]. Moreover, one secretome analysis of human autopsy samples showed reduced vinculin levels in atherosclerotic coronary artery lesions [24]. These findings thus suggest that the down-expression of vinculin in atherosclerotic lesions would contribute to the progression of atherosclerosis. However, Zhong et al. [25] demonstrated that oxidized LDL-treated human umbilical endothelial cells displayed a vinculin increase in total cell content with a reduction in membrane fraction and an increase in cytosol content, and that high shear stress increased vinculin membrane fraction and reduced focal adhesion. A further study is still needed to clarify the precise mechanism and role of vinculin in the progression of atherosclerosis.

Kim et al. [26] reported vinculin concentrations in plasma to be higher in 133 subjects with age-related macular degeneration than in 100 healthy subjects. Another study of proteomics showed that serum vinculin concentrations were related to 5-year mortality in 2473 elderly men [27]. Regarding blood vinculin concentrations and atherosclerotic diseases, Pan et al. [28] performed proteomics of plasma in 12 patients with acute myocardial infarction (AMI) and reported vinculin concentrations to be high in AMI patients. They suggested that high vinculin concentrations in blood may be a result of myocardial damage. Wang et al. [29] conducted a proteomics analysis of serum in 30 patients with aortic dissection, 30 with AMI, and 30 healthy controls. Vinculin concentrations in patients with aortic dissection but not in those with AMI were higher than in the controls. They suggested that vinculin could be a biomarker for aortic dissection. Vinculin is a cytoplasmic protein that exists mainly at the cell–ECM and cell–cell adhesions, and vinculin exists as the active form in the focal adhesions, whereas it exists as the inactive form in the cytoplasm [30]. The mechanism of how vinculin is secreted from the cells into the blood remains unclear. Considering the localization and function of vinculin, vinculin in the blood is considered to be due to tissue leakage [26,31]. López-Farré et al. [32] reported the down-expressed vinculin in platelets from 16 patients with ACS. However, Kristensen et al. [31] performed proteomics of plasma from 30 subjects with no coronary calcium, 30 with coronary calcium, and 30 with ACS, using computed tomography (CT). Vinculin concentrations in ACS patients were significantly higher than in those with no coronary calcium, but there was no marked difference in vinculin concentrations between subjects with and without calcium. One small study also reported no significant difference in plasma vinculin concentrations in 27 patients undergoing CABG and 35 healthy subjects [15]. Our present study was conducted to elucidate plasma vinculin concentrations in patients with stable CAD and to show whether or not vinculin concentrations can be a biomarker for CAD. We evaluated plasma vinculin concentrations in 327 patients undergoing coronary angiography by ELISA with the same kit used in the study by Pan et al. [28]. However, what we found was that vinculin concentrations were not markedly different between 177 patients with CAD and 150 without CAD and did not correlate with CAD severity. Vinculin concentrations did not correlate with talin-1 or CRP concentrations. As we previously reported [17], talin-1 concentrations in patients with stable CAD were high and correlated with CAD severity. Thus, plasma vinculin concentrations are unlikely to be a biomarker for CAD.

For clinical practice, the measurement of troponins I and T concentrations in blood is usually used to detect myocardial necrosis and to diagnose AMI. However, in addition to patients with AMI, some patients with COVID-19 infection can be troponin I positive [33]. Even healthy children and adolescents can also be troponin I and T positive after exercise [34]. Moreover, some differences in the predictive abilities of future events and the upstream genetic determinants between troponin I and T were recently reported. Welsh et al. [35] demonstrated that elevated troponin I concentrations were closely related to cardiovascular outcomes, whereas elevated troponin T concentrations were closely related to non-cardiovascular death. They also reported that the loci of troponin I but not troponin T presented near the vinculin encoding site, possibly affecting the cytoskeletal modeling in diseased cardiac tissue. Vinculin is a costametric protein in cardiomyocytes [36], and vinculin overexpression in myocardium was reported to be associated with aging and heart failure [37]. However, because most of our study patients with stable CAD did not have any measurements of troponin I or T concentrations, we could not provide the information on the correlations between vinculin concentrations and troponin I or T concentrations. Moreover, because our present study had no patients with ACS, a further study in ACS patients is needed to clarify the role and prognostic value of vinculin in ACS.

Talin-1 is a potent integrin activator affecting cell adhesion and apoptosis [1,4]. Both vinculin and talin-1 were shown to be down-expressed in atherosclerotic lesions [17], but a proteomics analysis showed high plasma talin-1 concentrations in patients with lacunar infarction and recurrence [38]. Another proteomics study showed no difference in talin-1 concentrations between 30 subjects with coronary calcium and 30 without it by CT [31]. However, we reported that plasma talin-1 concentrations were higher in patients with CAD than without CAD and correlated with CAD severity and CRP concentrations. Inflammatory responses are activated by ATP, and talin-1 is secreted exosomally from macrophages by ATP [39]. Since inflammation plays a role in atherosclerosis [40], high plasma talin-1 concentrations in CAD patients may reflect inflammation and atherosclerosis in the coronary arteries.

Our study had some limitations. First, coronary atherosclerosis was diagnosed by angiography. It cannot see plaques and shows only lumen characteristics. Although CAD was angiographically proven in 177 (54%) of the 327 study patients, intravascular ultrasound, which visualizes plaques, was not always performed. Moreover, fractional flow reserve was not always measured to evaluate functional stenosis. Second, vinculin and talin-1 exist in almost all tissues, including cardiac and vascular smooth muscle cells [1,10,17]. However, because we measured vinculin concentrations only in peripheral blood but not in the coronary sinus, we could not show the main resources of plasma vinculin in patients. Third, most of the previous studies reporting plasma vinculin concentrations used traditional iTRAQ [26,27,28] or new label-free [29] proteomics analyses. Such analyses are recognized to be a reliable and sensitive method for detecting biomarkers [29,41], but they were not available in our study. Therefore, we measured plasma vinculin concentrations using an ELISA method with a commercially available kit. Fourth, in our study, of the 327 study patients, 177 (54%) had CAD, and 147 (45%) showed talin-1 concentration > 0.28 ng/mL. Our study’s sample size was considered adequate to detect CAD with high talin-1 (>0.28 ng/mL) by statistical power of 80% and α-value of 0.05, since 307 patients were determined to be an adequate size. However, the relatively small number of study patients is a study limitation. Fifth, because we recently reported high talin-1 concentrations in CAD patients [18] and the interaction of vinculin with talin-1 is important for focal adhesions, the present study extended our previous study [18] by measuring vinculin concentrations after its publication. As a result, the blood samples of 22 patients were insufficient, and vinculin concentrations were measured in the remaining 327 patients. This was a study limitation. Sixth, since our study was cross-sectional, it could not establish causality and solely shows associations. Moreover, a proteomics study reported vinculin concentrations to be related to 5-year mortality in elderly [26]. To elucidate the prognostic values of vinculin concentrations in CAD patients, a further study prospectively is needed. Finally, we had no healthy controls. Even patients without angiographic CAD may have some atherosclerosis in the coronary arteries.

## 5. Conclusions

Plasma vinculin concentrations in patients with CAD were not high and were not associated with the presence or severity of CAD. No correlation was found between vinculin and taliin-1 or CRP concentrations. Vinculin concentrations are unlikely to reflect atherosclerosis and inflammation in coronary arteries.

## Figures and Tables

**Table 1:** Clinical characteristics of CAD(-) and CAD groups.

	CAD(-) (*n* = 150)	*p*-Value CAD(-) vs CAD	CAD (*n* = 177)	1-VD (*n* = 79)	2-VD (*n* = 57)	3-VD (*n* = 41)	Among 4 Groups
Age (years) Sex (men) Hypertension Systolic BP (mmHg) Diabetes mellitus HbA1c (%) Smoking Hypercholesterolemia Statin use LDL-cholesterol (mg/dL) HDL-cholesterol (mg/dL) CRP (mg/L)	65 ± 12 91 (61%) 92 (61%) 129 ± 21 23 (15%) 6.0 ± 0.7 44 (29%) 65 (43%) 41 (27%) 114 ± 28 59 ± 14 0.43 [0.21, 0.90]	<0.001 0.005 0.002 0.117 <0.001 0.002 0.033 0.013 0.001 0.641 <0.001 0.001	70 ± 9 134 (76%) 137 (77%) 133 ± 18 59 (33%) 6.3 ± 0.9 72 (41%) 101 (57%) 80 (45%) 112 ± 29 52 ± 14 0.58 [0.32, 1.43]	69 ± 10 58 (73%) 59 (75%) 131 ± 16 22 (28%) 6.2 ± 0.8 35 (44%) 43 (54%) 34 (43%) 108 ± 28 56 ± 15 0.58 [0.32, 1.38]	69 ± 10 42 (74%) 43 (75%) 137 ± 20 22 (39%) 6.4 ± 1.1 25 (44%) 33 (58%) 25 (44%) 115 ± 31 51 ± 12 0.56 [0.30, 1.11]	72 ± 8 34 (83%) 35 (85%) 130 ± 18 15 (37%) 6.2 ± 0.9 12 (29%) 25 (61%) 21 (51%) 117 ± 26 48 ± 13 0.81 [0.42, 2.21]	<0.001 0.020 0.009 0.073 0.001 0.003 0.055 0.085 0.008 0.301 <0.001 0.004

## Data Availability

Data that support our findings can be requested from the corresponding author.
